# A distinct gene expression mechanism for d‐amino acid utilization in hyperthermophilic archaeon *Pyrococcus horikoshii*

**DOI:** 10.1111/febs.70545

**Published:** 2026-04-16

**Authors:** Ryushi Kawakami, Tomoki Kawase, Taira Uehara, Haruhiko Sakuraba, Tomohiko Matsuzawa, Junji Hayashi, Toshihisa Ohshima

**Affiliations:** ^1^ Division of Bioscience and Bioindustry, Graduate School of Technology, Industrial and Social Sciences Tokushima University Japan; ^2^ Division of Bioresource Science, Graduate School of Sciences and Technology for Innovation Tokushima University Japan; ^3^ Department of Applied Biological Science, Faculty of Agriculture Kagawa University Japan; ^4^ Department of Biomedical Engineering, Faculty of Engineering Osaka Institute of Technology Japan

**Keywords:** amino acid racemase, archaea, d‐amino acid, *Pyrococcus horikoshii*, transcription factor

## Abstract

Growing the hyperthermophilic archaeon *Pyrococcus horikoshii* OT‐3 in medium supplemented with d‐*allo*‐Ile instead of l‐Ile markedly upregulates the activity of broad substrate specificity amino acid racemase (BAR). In *P. horikoshii* genome, the BAR gene (*PH0138*) forms a cluster with *PH0137*, which encodes a putative transporter protein, and *PH0140* that encodes a feast/famine regulatory protein (FFRP), involved in the transcriptional regulation of metabolic pathway genes. Here, we performed gene expression, protein–DNA interaction, and crystallographic analyses to elucidate the expression mechanism of the BAR cluster. Gene expression analysis revealed that d‐*allo*‐Ile simultaneously induces *PH0138* and *PH0137*. Electrophoretic mobility shift assays demonstrated that the PH0140 protein binds to the *PH0137* promoter in the presence of l‐Ile, but this interaction is disrupted by d‐*allo*‐Ile, identifying PH0140 as a d‐amino acid‐responsive regulatory protein (DARP). The crystal structures of DARP bound to l‐Ile and d‐*allo*‐Ile were compared with those of another FFRP family member, *P. horikoshii* FL11. Our findings reveal a dimeric arrangement of l‐Ile‐bound DARP resembling the DNA‐bound (open) form of FL11, whereas d‐*allo*‐Ile‐bound DARP corresponds to the DNA‐unbound (closed) form. These conformational changes result from subtle alterations in hydrogen bonding around the coregulators. Furthermore, cultivation in d‐*allo*‐Ile substitution medium only impacted the expression of *PH0138* and *PH0137*, and not the other genes, suggesting that DARP regulates a more limited gene set than FL11. Cumulatively, these results reveal a distinct mechanism by which an FFRP homolog controls d‐amino acid utilization.

## Graphical abstract

The genes *PH0137*, *PH0138*, and *PH0140* form a cluster in the genome of *Pyrococcus horikoshii*. The *PH0140* gene encodes a transcription factor identified as a d‐amino acid‐responsive regulatory protein (DARP). In feast mode, l‐Ile‐bound DARP binds to the promoter and represses transcription of *PH0137* and *PH0138*. In famine mode, DARP binds to d‐*allo*‐Ile and dissociates from the promoter, thereby activating transcription of these genes.

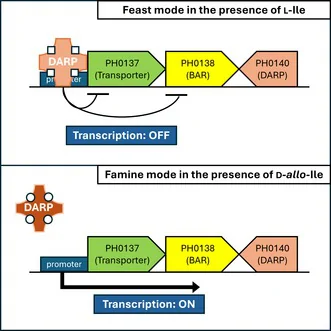

**Abbreviations**
BAR: broad substrate specificity amino acid racemase
DARP: d‐amino acid‐responsive regulatory protein
EMSA: electrophoretic mobility shift assay
FFRP: feast/famine regulatory protein
PDB: protein data bank
RMSD: root‐mean‐square deviation
PLP: pyridoxal 5'‐phosphate
RT‐qPCR: quantitative reverse transcription PCR
Tb: thrombin
TPM: transcripts per million

## Introduction

d‐Amino acids have been shown to have diverse physiological functions *in vivo*. In humans, d‐Ser and d‐Asp are associated with higher brain functions and the regulation of hormone synthesis, respectively [1, 2]. d‐Ala contributes to osmotic pressure regulation as an osmolyte in aquatic crustaceans [3, 4]. Additionally, d‐Ala and d‐Glu are essential constituents of bacterial cell wall peptidoglycan [5, 6]. Various d‐amino acids are synthesized by fermentative microorganisms, particularly lactic acid bacteria [7, 8]. Additionally, an intestinal environmental maintenance system has been proposed that involves d‐amino acids produced by gut microbiota [9].

A range of free d‐amino acids has also been detected in extracts from archaeal cells. Notably, *Thermoplasma acidophilum*, *Thermococcus* spp. KS‐1, KS‐8, *Pyrococcus* spp. OII, GB‐D, *Desulfurococcus* sp. SY, and *Halobacterium salinarum* exhibited high proportions (13–50%) of d‐Asp, d‐Ala, d‐Leu, and d‐Lys relative to their respective total amino acid contents [10, 11]. Free d‐amino acids within these cells are predominantly generated via pyridoxal 5′‐phosphate (PLP)‐dependent or PLP‐independent racemization of their corresponding l‐isomers by amino acid racemases. Both PLP‐dependent (Asp and Ser racemases) and PLP‐independent (Asp and Pro racemases) enzymes have been identified and characterized in archaeal cells [12, 13, 14, 15]. Furthermore, dehydrogenase activities related to d‐amino acid catabolism including d‐Ala, d‐Asp, d‐Ser, and d‐Pro have been identified in *Pyrobaculum islandicum* [16, 17]. Indeed, genes encoding various d‐amino acid metabolizing enzymes are frequently present in archaeal genomes; however, the specific physiological role of d‐amino acids in archaeal cells remains unclear.

*Pyrococcus horikoshii* OT‐3 is a hyperthermophilic archaeon isolated from a hydrothermal vents [18]. Recently, we investigated the l‐amino acid requirement and d‐amino acid utilization for *P. horikoshii* growth, reporting growth on media supplemented with d‐amino acid (Leu, Ile, Val, Met, Phe, Trp, Tyr, or Arg) instead of the respective l‐isomer [19]. Novel PLP‐dependent amino acid racemase activity toward Met, Leu, and Phe was also identified in the crude extract of *P. horikoshii*, leading to the identification and cloning of the enzyme gene (ORF ID: *PH0138*) and characterization of the recombinant protein [19, 20]. This enzyme, termed broad substrate specificity amino acid racemase (BAR), forms a dimeric structure with subunit molecular masses of 52 kDa and exhibits activity toward ten amino acids, including Met, Leu, Ile, and Phe (25.9–65.7 μmol/min/mg for the l‐amino acid‐forming reaction). BAR activity in the crude extract of *P. horikoshii* cells grown in l‐Ile‐deficient medium supplemented with d‐*allo*‐Ile is 34 times higher than in cells grown in standard medium. Meanwhile, BAR activity is not upregulated in the presence of Met, Leu, Phe, Val, Trp, Tyr, or Arg [19]. These findings collectively indicate that BAR facilitates the utilization of d‐amino acids for *P. horikoshii* growth.

Within the *P. horikoshii* genome, the BAR gene (*PH0138*) is positioned downstream of *PH0137*, which encodes a putative Ykvl family transporter. Directly downstream, in the opposite orientation, lies *PH0140*, which encodes a feast/famine regulatory protein (FFRP), identified as FL5 [21]. Together, these three genes comprise the BAR cluster (Fig. 1).

**Fig. 1:**
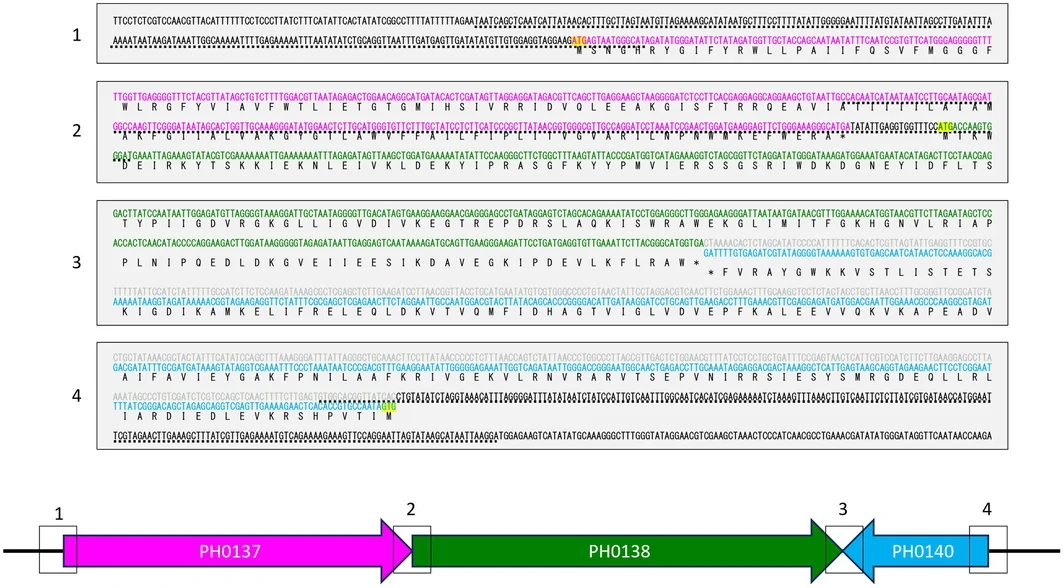
Structure of the broad substrate specificity amino acid racemase (BAR) gene cluster. *PH0137*, *PH0138*, and *PH0140* are represented by the magenta, green, and cyan arrows, respectively, and detailed sequence information in the boxed regions (1–4) was shown in the upper panels. DNA sequences of *PH0137* and *PH0138* are shown in magenta and green, respectively. *PH0140* is encoded on the complementary strand and is shown in cyan letters. Start codons (ATG or GTG) for each gene are depicted in yellow; amino acid sequences of these genes are aligned under their DNA sequences. The sequences of the DNA probes used for electrophoretic mobility shift assays are underlined with the dashed lines. The genomic sequence within the BAR gene cluster was obtained from the Kyoto Encyclopedia of Genes and Genomes database (https://www.kegg.jp/kegg/). The alignment was generated using the Genetyx program (nihon server, Tokyo, Japan).

In the current study, the enzymatic activity and gene expression of BAR were analyzed under various growth conditions, revealing that gene expression is regulated by PH0140 protein binding to the *PH0137* promoter in the presence of l‐Ile—a process inhibited by d‐*allo*‐Ile. Cumulatively, the results suggest that the PH0140 protein functions as a d‐amino acid‐responsive regulatory protein (DARP). Furthermore, the crystal structures of l‐Ile‐ and d‐*allo*‐Ile‐bound DARP were determined and their coregulator‐binding modes compared. This work presents a novel gene expression mechanism in archaea mediated by an FFRP homolog that is responsive to d‐ and l‐amino acid isomers.

## Results

### Determination of BAR activity under various cultivation conditions

The BAR activity in the crude extracts from *P. horikoshii* cells grown under various cultivation conditions was determined using a spectrophotometric assay with l‐Met as substrate. In addition to previous results [19], substitution of l‐amino acids such as Ala, Asn, Asp, Gln, Glu, His, Lys, Pro, Ser, or Thr in the l‐amino acid medium (containing all 20 l‐amino acids) with the corresponding d‐isomer did not increase BAR activity. This suggests that BAR activity increased only when l‐Ile was replaced by d‐*allo*‐Ile in the medium.

The time‐dependent increase in BAR activity was investigated by adding d‐*allo*‐Ile to the medium after *P. horikoshii* cells had been cultivated for 20 h in the absence of l‐Ile. An increase in BAR activity was observed within 1 h and continued for at least 12 h (Fig. 2A). In contrast, simultaneous addition of l‐Ile and d‐*allo*‐Ile did not increase BAR activity (Fig. 2B).

**Fig. 2:**
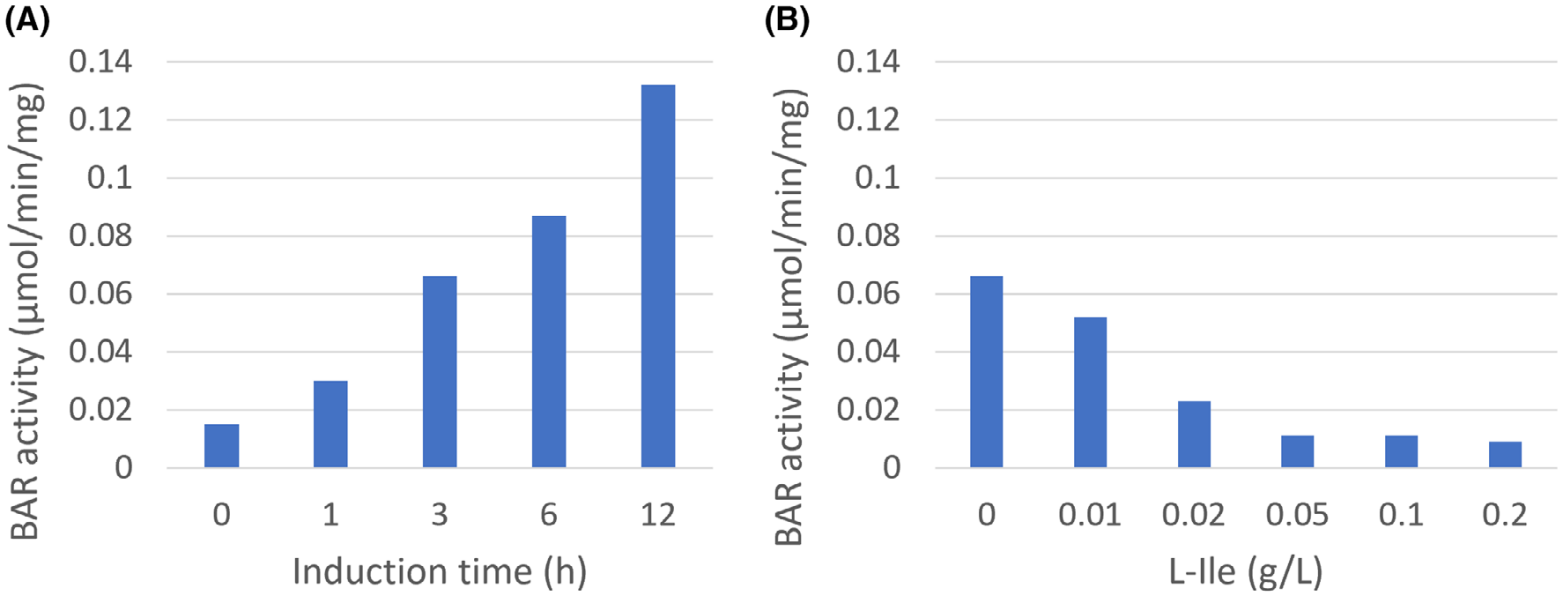
Broad substrate specificity amino acid racemase (BAR) activity in crude extracts under different cultivation conditions. (A) After cultivation for 20 h in the absence of l‐Ile, 0.1 g/L of d‐*allo*‐Ile was added to the medium. After an additional 0–12 h, BAR activity in crude extracts was measured in triplicate. Data were obtained from a single experiment (*n* = 1). (B) After cultivation for 20 h without l‐Ile, 0.1 g/L d‐*allo*‐Ile and various amounts of l‐Ile (0–0.2 g/L) were added to the medium at the same time. After 3 h of further cultivation, BAR activity in the crude extract was determined in triplicate. Data were obtained from a single experiment (*n* = 1).

### Gene expression analyses by RNA‐seq and quantitative reverse transcription PCR (RT‐qPCR)

Total RNA was extracted from cells cultivated for 24 h in l‐amino acid medium containing 20 l‐amino acids or in the d‐*allo*‐Ile substitution medium—l‐Ile in the l‐amino acid medium was replaced with d‐*allo*‐Ile. After extracting mRNA, the synthesized cDNAs were sequenced using NextSeq 500, and gene expression levels were evaluated using transcripts per million (TPM) values. The expression level of the *PH0138* gene increased significantly from 23.3 TPM in l‐amino acid cultivation to 92.7 TPM in d‐*allo*‐Ile substitution cultivation (~4‐fold; Table 1). The adjacent *PH0137* gene also showed a 6‐fold increase in expression from 12.1 to 71.8 TPM. These results suggest that d‐*allo*‐Ile simultaneously induced both genes, which likely function as an operon. On the other hand, the expression level of the *PH0140* gene slightly decreased from 56.4 to 33.7 TPM. No significant changes were observed in the expression of other genes encoding functional proteins after adding d‐*allo*‐Ile (Table 1).

**Table 1:** Analysis of differentially expressed genes by RNA‐seq.

Gene ID	Fold change at TPM (d‐*allo*‐Ile/l‐amino acid)	Product
PH0137	5.95	378 aa membrane protein
PH0138	3.97	466 aa amino acid racemase (BAR)
PH0218	2.79	140 aa hypothetical protein
PH0975	2.75	110 aa hypothetical protein
PH0217	1.77	106 aa hypothetical protein
PH0055	1.44	103 aa hypothetical protein
PH1759	1.43	150 aa 50S ribosomal protein L19
PH1504	1.35	107 aa hypothetical protein
PH1998	1.26	111 aa 50S ribosomal protein L12
PH1501	1.22	438 aa amino acid racemase (MAR)
PH0563	1.17	330 aa hypothetical protein
PHS046	1.13	70 aa archaeal histone
PH1940	1.11	109 aa 50S ribosomal protein L44
PH0192	1.04	129 aa hypothetical protein

*Note:* After removal of the genes that were less than 10 of the total counts in RNA‐seq analysis, the genes that showed more than 1.0‐fold higher expression at TPM values in d‐*allo*‐Ile substitution cultivation than in l‐amino acid cultivation are listed.

The effects of d‐*allo*‐Ile on the expression of *PH0137*, *PH0138*, and *PH0140* were further investigated by RT‐qPCR. Expression of *PH0137* and *PH0138* was induced within 0.5 h after adding d‐*allo*‐Ile to the culture; their expression levels increased by more than 10‐fold within the first hour compared to those cultivated without d‐*allo*‐Ile (Fig. 3, Conditions 1–2). Although expression levels decreased slightly (condition 3), the genes remained overexpressed by 4–6 times at 8 h (unpublished data) and 24 h (RNA‐seq data) after induction. In contrast, no such induction was observed when l‐Ile was added after d‐*allo*‐Ile (Fig. 3, Condition 4) or when d‐*allo*‐Ile and l‐Ile were added simultaneously (Fig. 3, Condition 5). These results suggest that d‐*allo*‐Ile induces the expression of both genes, whereas l‐Ile suppresses their expression. RT‐qPCR experiments revealed no change in *PH0140* expression (Fig. 3).

**Fig. 3:**
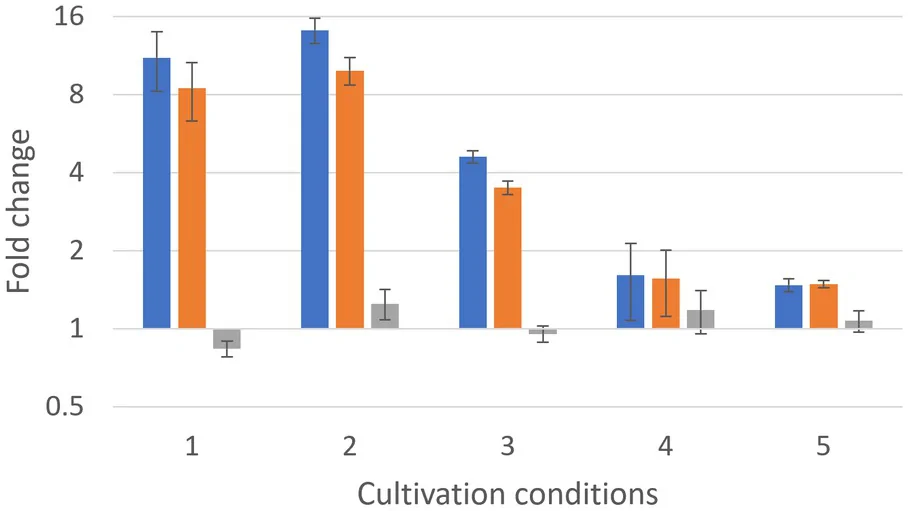
Gene expression analysis by qPCR under various cultivation conditions. In all conditions, *P. horikoshii* were pre‐cultivated for 20 h without l‐Ile (control condition). Conditions 1–3: after adding 0.1 g/L of d‐*allo*‐Ile to the medium, cultivation continued for 0.5, 1.0, and 2.0 h. Condition 4: after 0.5 h cultivation with 0.1 g/L of d‐*allo*‐Ile, 0.1 g/L l‐Ile was added, and cultivation was continued for another 0.5 h. Condition 5: l‐Ile and d‐*allo*‐Ile were added simultaneously, and cultivation continued for 0.5 h. After cultivation under each condition, total RNAs were extracted and used for cDNA synthesis. *PH0137* (blue), *PH0138* (orange), and *PH0140* (gray) were amplified as the target genes. The 23S rRNA gene was used to normalize gene expression, and the fold change in each condition relative to the control was calculated using the comparative threshold cycle method (ΔΔCt). The experiments were conducted in triplicate (*n* = 3). Data represent as means ± SEM.

### Purification of DARP and digestion by thrombin (Tb)

Initially, the expression vector, pET11a/*PH0140* was constructed, and untagged proteins were used for the electrophoretic mobility shift assay (EMSA) and crystallographic analysis. Heat treatment of the crude extract resulted in a distinct protein band at approximately 18 kDa (Fig. 4A, lanes 1 and 2), demonstrating efficient production of the untagged DARP protein in *Escherichia coli*. While certain DARP was bound to the DEAE Sepharose column, a substantial portion did not (Fig. 4A, lanes 3 and 4). However, post‐purification analysis revealed that *E. coli* DNA fragments contaminated the untagged DARP (Fig. 4B), and neither ammonium sulfate precipitation nor streptomycin sulfate treatment removed these contaminants. Thus, His‐tagged proteins were used for subsequent analyses.

**Fig. 4:**
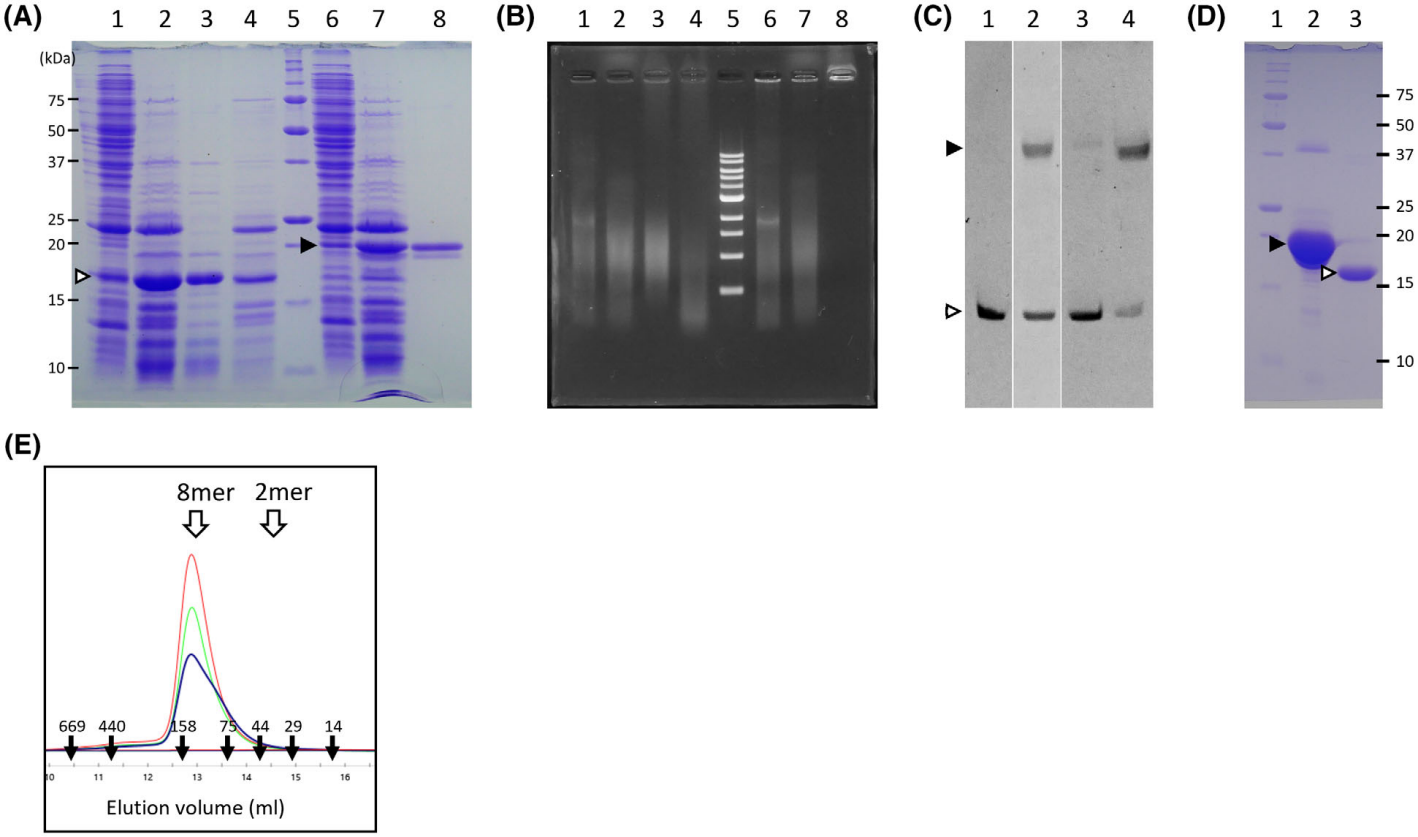
Purification and electrophoretic mobility shift assay (EMSA) analysis of d‐amino acid‐responsive regulatory protein (DARP). SDS/PAGE (A) and agarose gel electrophoresis (B) of the untagged and His‐tagged DARP at each purification step. Lane 1–4: crude extract, heat treatment, and flow through and elution fractions of DEAE‐Cellurofine of untagged DARP; lane 5: markers; lane 6–8: crude extract, heat treatment, and elution fraction of Ni^2+^‐chelating Sepharose of His‐tagged DARP. Untagged and His‐tagged DARP are indicated by the white and black arrows, respectively. A representative result from a single experiment is shown (*n* = 1). (C) EMSA of untagged, His‐tagged, and thrombin (Tb)‐digested DARP with 10 nm of *PH0137* DNA probe in the presence of 1 mm l‐Ile. Lane 1: without proteins; lane 2: 50 nm untagged DARP; lane 3: 100 nm His‐tagged DARP; and lane 4: 100 nm Tb‐digested DARP. The positions of free and DARP‐bound probes are indicated by the white and black arrows, respectively. Lane 2 is obtained from a different gel than lanes 1, 3, and 4. The lanes are assembled for presentation, and gaps indicate the boundaries between the gels. No other modifications are made to the images. A representative result from a single experiment is shown (*n* = 1). (D) SDS/PAGE analysis of His‐tagged and Tb‐digested DARP. Lane 1: markers; lane 2: His‐tagged DARP; and lane 3: Tb‐digested DARP. His‐tagged and Tb‐digested DARP are indicated by the black and white arrows, respectively. A representative result from a single experiment is shown (*n* = 1). (E) Elution profiles of the DARP on an SEC650 gel filtration column. The proteins were eluted with 10 mm Tris/HCl (pH 8) and 0.5 m NaCl at a flow rate of 1.0 mL/min (blue). Buffers containing 10 mm l‐Ile (red) and 10 mm d‐*allo*‐Ile (green) were used. The molecular masses of standards (kDa) and oligomeric states of the proteins estimated from the elution peaks are indicated in the profile. A representative result from a single experiment is shown (*n* = 1).

His‐tagged DARP (approximately 20 kDa) was overexpressed in *E. coli* transformed with pET15b/*PH0140* vector and purified His‐tagged DARP without DNA contaminants was obtained by heat treatment and Ni^2+^‐chelating Sepharose purification (Fig. 4A,B). Preliminary EMSA demonstrated DNA‐binding activity with the untagged DARPs, whereas the His‐tagged variants lacked this activity (Fig. 4C, lanes 1 and 2). These results suggested that the N‐terminal His‐tag sequence (20 amino acids) inhibited DARP's DNA‐binding activity. To address this, the His‐tag was removed by Tb digestion, yielding Tb‐digested DARP with three additional amino acids (GSH), which restored DNA‐binding activity comparable to untagged form (Fig. 4C,D). The molecular mass of Tb‐digested DARP was determined to be 113–124 kDa using an SEC650 gel filtration column (Bio‐Rad, Tokyo, Japan) in the absence and presence of l‐Ile or d‐*allo*‐Ile (Fig. 4E), indicating that its oligomeric structure remained unaffected by ligand type.

### Interaction between DNA probe and DARP

For EMSA, a region spanning −200 bp to +15 bp relative to the start site of each gene (*PH0134, PH0137, PH0138*, and *PH0140*) was amplified by PCR and used as a DNA probe. The *PH0134* gene was selected as the negative control.

When Tb‐digested DARP and DNA probes were mixed in the presence or absence of l‐Ile and incubated at 70 °C for 15 min, protein–DNA interactions were observed only with the *PH0137* probe in the presence of l‐Ile (Fig. 5A). Next, we examined the protein–DNA interactions at various concentrations of l‐Ile and d‐*allo*‐Ile. Equivalent band shifts were detected within the 0.1–1.0 mm l‐Ile range (Fig. 5B). In the presence of a fixed concentration of d‐*allo*‐Ile (0.5 mm and 1.0 mm), the band shift gradually disappeared as l‐Ile concentration decreased (Fig. 5B), though non‐specific bands appeared. These results indicate that in the presence of d‐*allo*‐Ile, the DARP protein does not interact with the *PH0137* probe and that d‐*allo*‐Ile counteracts the interaction observed with l‐Ile.

**Fig. 5:**
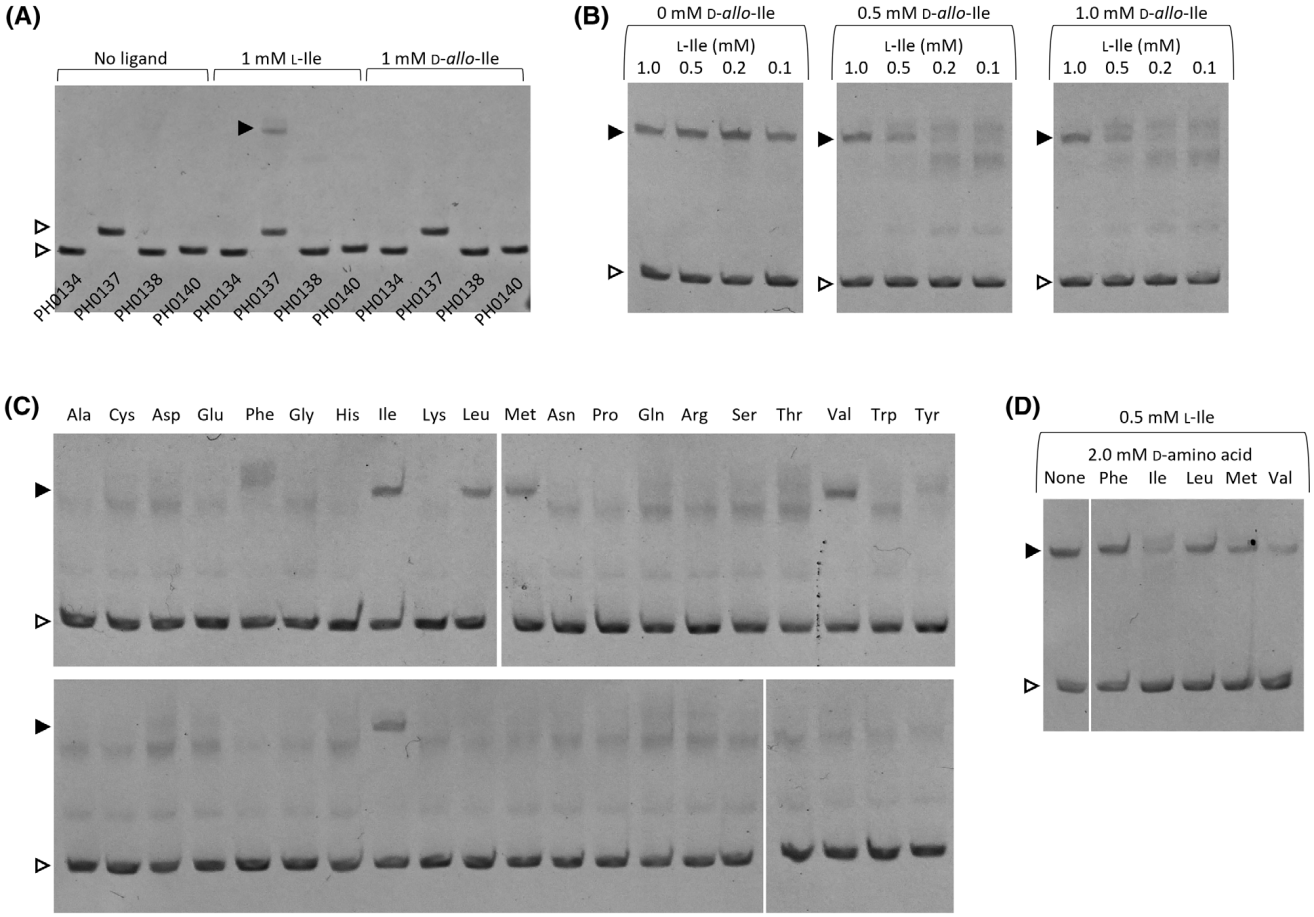
Interaction of d‐amino acid‐responsive regulatory protein (DARP) with DNA probes in the presence and absence of d‐ or l‐amino acids. (A) 25 nm thrombin (Tb)‐digested DARP incubated with 10 nm DNA probe (*PH0134*, *PH0137*, *PH0138*, or *PH0140*) in the presence of 1 mm l‐Ile or 1 mm d‐*allo*‐Ile. (B) 50 nm Tb‐digested DARP incubated with 10 nm *PH0137* DNA probe at varying concentrations of l‐Ile and d‐*allo*‐Ile. (C) 50 nm Tb‐digested DARP incubated with 10 nm *PH0137* DNA probe in the presence of different l‐amino acids at 1 mm in the absence (upper panel) and presence (lower panel) of 1 mm of d‐*allo*‐Ile. (D) 50 nm Tb‐digested DARP incubated with 10 nm *PH0137* DNA probe at 2.0 mm of the indicated d‐amino acids in the presence of 0.5 mm l‐Ile. For panels C and D, the gaps were added between the different gels. For all panels, the positions of free and DARP‐bound probes are indicated by the white and black arrows, respectively. A representative result from a single experiment is shown (*n* = 1).

The interaction between DARP and the *PH0137* probe was examined in the presence of various l‐amino acids at 1 mm. Specific band shifts were observed for l‐Ile, l‐Leu, l‐Met, and l‐Val at identical positions but not for other l‐amino acids, namely Ala, Cys, Asp, Glu, Gly, His, Lys, Asn, Pro, Gln, Arg, Ser, Thr, Trp, and Tyr (Fig. 5C). With the exception of l‐Ile, these band shifts disappeared after the addition of 1 mm d‐*allo*‐Ile (Fig. 5C). The indistinct bands observed during interaction with other amino acids were interpreted as non‐specific. Furthermore, in the presence of l‐Ile, the band shift nearly disappeared with the addition of d‐*allo*‐Ile and d‐Val, but only slightly with d‐Leu and d‐Met (Fig. 5D). These results suggest that several l‐ and d‐amino acids are involved in the interaction between DARP and the *PH0137* probe, with l‐Ile and d‐*allo*‐Ile being the most effective coregulators.

In addition to these amino acids, the interaction between DARP and the *PH0137* probe was assessed with several non‐canonical amino acids: nor‐leucine, nor‐valine, and 2‐aminobutyrate. Band shifts were observed with l‐nor‐leucine and l‐nor‐valine, but not with l‐2‐aminobutyrate (data not shown). When l‐Ile was present, d‐nor‐leucine and d‐nor‐valine slightly reduced band shifts, similar to the effects of d‐Leu and d‐Met, whereas d‐2‐aminobutyrate did not have this effect. Given that the potential existence of these compounds in deep‐sea environments is currently unknown, these results can be considered part of ligand specificity.

### Overall structure of DARP

His‐tagged DARP was used to determine the crystal structure. The structure of l‐Ile‐bound DARP was refined at a resolution of 1.39 Å (Table 2). The protein was then assembled into an octamer (Fig. 6). The asymmetric unit comprised one subunit with a solvent content of 50.8%, corresponding to a Matthews coefficient [22] of 2.5 Å^3^ Da^−1^. The model contained 10–158 ordered amino acid residues, one l‐Ile molecule, two sulfate ions, and 129 water molecules. The N‐terminal His‐tag sequence and residues 1–9 were disordered and not visible in the electron density map.

**Table 2:** Data collection and refinement statistics.

	l‐Ile‐bound DARP	d‐*allo*‐Ile‐bound DARP
PDB code	9W4W	9W4V
Data collection		
Synchrotron light source	Photon Factory	Photon Factory
Beamline	AR‐NW12A	AR‐NE3A
Wavelength (Å)	1.0	1.0
No. of frames	180	180
Oscillation width (deg)	1.0	1.0
Detector distance (mm)	141.68	257.1
Exposure per frame (s)	1	1
Temperature (K)	100	100
Indexing and scaling		
Space group	*I*422	*I*222
Unit cell parameters		
a (Å)	101.1	68.3
b (Å)	101.1	102.7
c (Å)	70.4	102.9
Resolution range (Å)^a^	50–1.39 (1.41–1.39)	50–2.2 (2.27–2.2)
Total No. of reflections	443 326	122 361
No. of unique reflections	36 972	18 772
Redundancy^a^	12.0 (11.4)	6.5 (6.7)
Completeness (%)^a^	100 (100)	100 (100)
< *I*/*σ*(*I*) >^a^	8.3 (2.0)	12.7 (1.7)
*R*_pim_ ^a^, ^b^	0.021 (0.30)	0.044 (0.65)
CC_1/2_ ^a^, ^c^	1.0 (0.805)	0.998 (0.671)
No. of chains per asymmetric unit	1	2
Refinement		
Resolution range (Å)	50–1.39	50–2.20
*R*/*R*_free_ (%)^a^, ^d^	17.8/19.5 (23.9/26.6)	19.8/22.9 (29.2/34.2)
No. of protein atoms	1203	2378
No. of water molecules	129	41
No. of ligands	l‐Ile, 1	d‐*allo*‐Ile, 2
	Sulfate ion, 2	Sulfate ion, 2
		Ethylene glycol, 2
Average B‐factors (Å^2^)	20.2	56.9
RMSD^e^		
Bond lengths (Å)	0.018	0.007
Bond angles (°)	1.9	1.4
Ramachandran statistics		
Favored (%)	98.7	98.6
Allowed (%)	1.3	1.4
Outliers (%)	0	0

^a^ Values in parentheses: highest resolution data shell.

^b^ *R*_pim_ = Σ_h_ [1/(/*n*_h_–1)]^1/2^ Σ_i_| < *I*_h_ > −I_h,i_|/Σ_h_ Σ_i_ *I*_h,I_, where *h* enumerates the unique reflections, *i* represents their symmetry‐equivalent contributors, and *n*_h_ denotes multiplicity.

^c^ CC_1/2_ = Correlation between intensities from random half‐data sets.

^d^ *R*_free_ calculated with randomly selected reflections (5%).

^e^ RMSD: root‐mean‐square deviation.

**Fig. 6:**
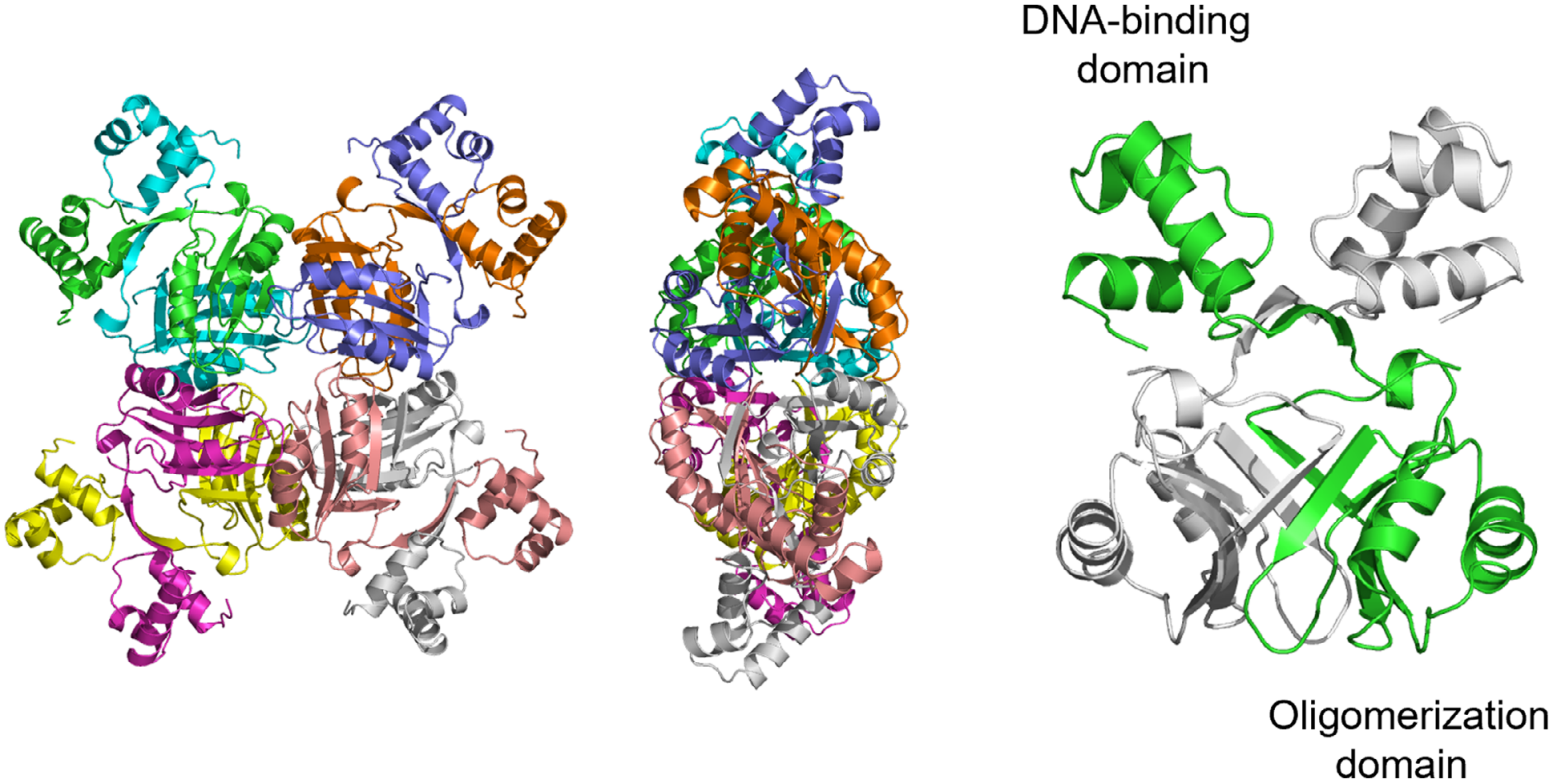
Overall structure of l‐Ile‐bound d‐amino acid‐responsive regulatory protein (DARP). Overall structure of the l‐Ile‐bound DARP octamer, with subunits shown in different colors (left). Subunits A–H are colored green, cyan, magenta, yellow, pink, gray, light blue, and orange, respectively. A rotated view is shown in the middle, and the dimer structure with subunits in different colors is shown on the right (Subunits A and B are shown in gray and green, respectively). The overall structure of the d‐*allo*‐Ile‐bound DARP octamer is highly similar to that of l‐Ile‐bound DARP octamer. Molecular graphics were generated with PyMOL (http://www.pymol.org/).

The structure of d‐*allo*‐Ile‐bound DARP was refined at a resolution of 2.20 Å (Table 2). The protein also assembled into an octamer, but the asymmetric unit comprised two subunits with a solvent content of 51.0%, corresponding to a Matthews coefficient of 2.5 Å^3^ Da^−1^. The model contained ordered amino acid residues 10–158 in both subunits, along with two d‐*allo*‐Ile molecules, two sulfate ions, two ethylene glycol molecules, and 41 water molecules. The N‐terminal His‐tag sequence and residues 1–9 were disordered and not visible in the electron density map. Although the overall structures of l‐Ile‐bound and d‐*allo*‐Ile‐bound DARP were highly similar, distinct conformational changes were observed (see below).

Upon submission of the l‐Ile‐bound DARP model to the DALI server [23] for identification of structurally similar proteins (as of July 9, 2025), the most closely related protein was FL11 from *P. horikoshii* (Protein Data Bank [PDB] code: 2ZNZ [l‐Lys‐bound structure]), with root‐mean‐square deviations (RMSD) of 1.4–1.6 [24]. The main‐chain coordinates of the DARP monomer were similar to those of FL11, including the dimeric arrangement of the octamer. The monomer folded into two domains: a smaller DNA‐binding domain and a larger oligomerization domain (Fig. 6).

For FL11, the octamer in the l‐Arg‐bound structure adopted a horseshoe shape with a gap (PDB code: 2ZNY), whereas the l‐Lys‐bound structure lacked this gap (PDB code: 2ZNZ) [24]. This difference was not observed between the l‐Ile‐bound and d‐*allo*‐Ile‐bound DARP structures, which exhibited highly symmetrical octamers, in which the four dimers formed a closed ring (Fig. 6).

### Coregulator‐binding site

The initial electron density map for l‐Ile‐bound DARP showed extra density at the dimer–dimer interface. After construction and refinement of the peptide chain, an l‐Ile molecule was modeled unambiguously into that density. Since l‐Ile was not present during crystal growth, this molecule may have originated in *E. coli* cells and was retained throughout protein purification. The map precisely defined the orientation of l‐Ile (Fig. 7A), with its carboxyl group forming hydrogen bonds with the side chains of Thr141 and Thr143 and the main‐chain NH of Thr143 and Gly108* (*: residue in the neighboring subunit [subunit G]). The α‐amino group of l‐Ile formed hydrogen bonds with the side chain of Asp111* and the main‐chain carbonyl groups of Val106*, Gly108*, and His110*. The side chain of l‐Ile formed a hydrophobic core, primary with Leu121, Ile128, Leu129, Val87*, and Ile105*.

**Fig. 7:**
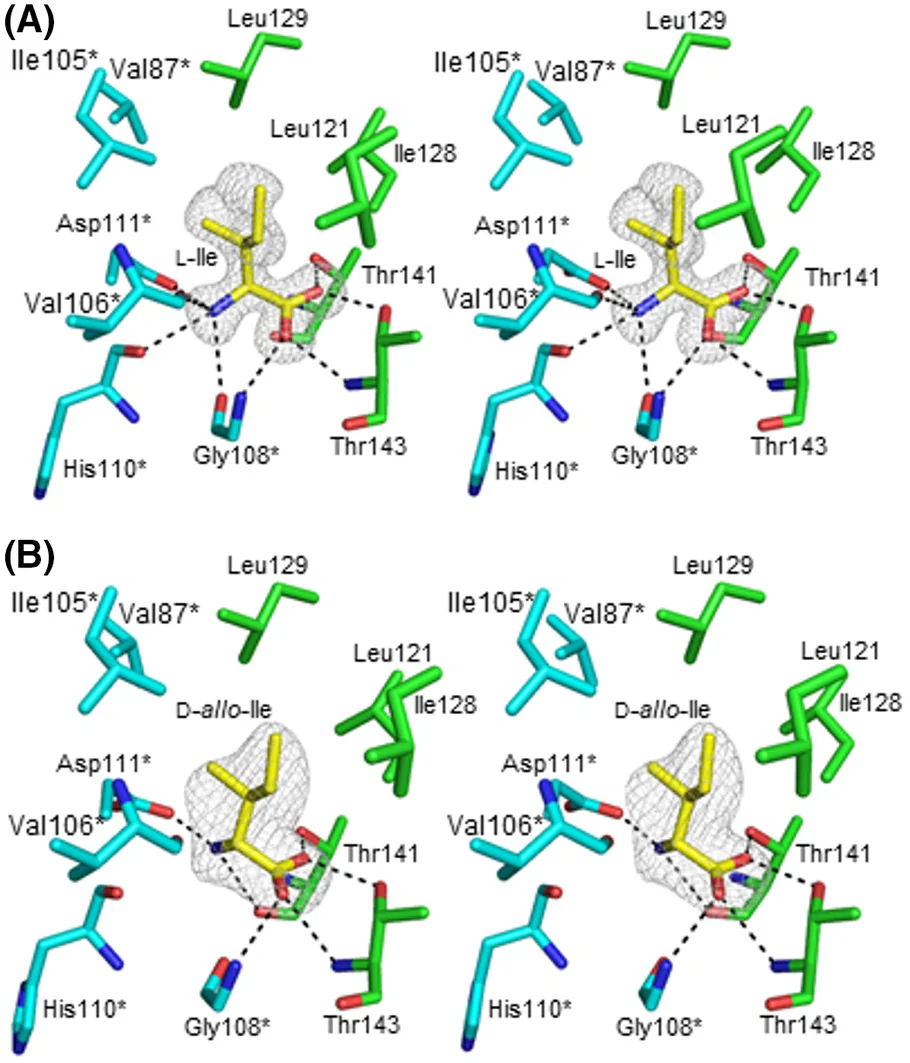
Stereographic close‐up of the coregulator‐binding site of l‐Ile‐bound d‐amino acid‐responsive regulatory protein (DARP) (A) and d‐*allo*‐Ile‐bound DARP (B). Residues that interact with coregulators in subunit A and in the neighboring subunit (subunit G) are shown in green and cyan, respectively. The l‐Ile and d‐*allo*‐Ile molecules are represented in yellow. The Fo‐Fc omit electron density map for the coregulator was generated using Polder Maps [25] (contoured at 3.5 σ). The hydrogen bond networks are shown as dashed lines. Molecular graphics were generated with PyMOL (http://www.pymol.org/).

In the initial electron density map of d‐*allo*‐Ile‐bound DARP, a clear electron density corresponding to the d‐*allo*‐Ile molecule was observed at the coregulator‐binding site. A comparison of the structures of the l‐Ile‐ and d‐*allo*‐Ile‐bound DARP revealed that the carboxyl group and side chain of d‐*allo*‐Ile occupied nearly the same positions as those of l‐Ile. The hydrogen bonds and hydrophobic interactions around these moieties in l‐Ile were conserved in the d‐*allo*‐Ile‐bound structure (Fig. 7B). In contrast, the positions of the α‐amino groups of the ligands differed between the two structures. The N atom of d‐*allo*‐Ile rotated clockwise around the Cα atom by approximately 83° relative to that of l‐Ile. As described above, all hydrogen bonds that stabilized the α‐amino group of l‐Ile were donated by residues in the neighboring subunit (subunit G). In contrast, the α‐amino group of d‐*allo*‐Ile formed a hydrogen bond with the main‐chain carbonyl of Thr141 in the same subunit (subunit A), in addition to interacting with the side chain of Asp111*. This replacement of hydrogen bonds may be responsible for the conformational change of the dimer as a functional unit (see below).

His‐tagged DARP failed to bind DNA regardless of the presence of l‐Ile in the EMSA reaction buffer. In contrast, Tb‐digested DARP did bind to DNA in the presence, but not in the absence of l‐Ile (Fig. 4C). Clear electron density corresponding to the l‐Ile ligand was observed in the His‐tagged DARP structure (Fig. 8A). However, preliminary structural analysis of Tb‐digested DARP revealed no electron density for l‐Ile (Fig. 8B), suggesting that l‐Ile dissociated from the protein following removal of the His‐tag sequence.

**Fig. 8:**
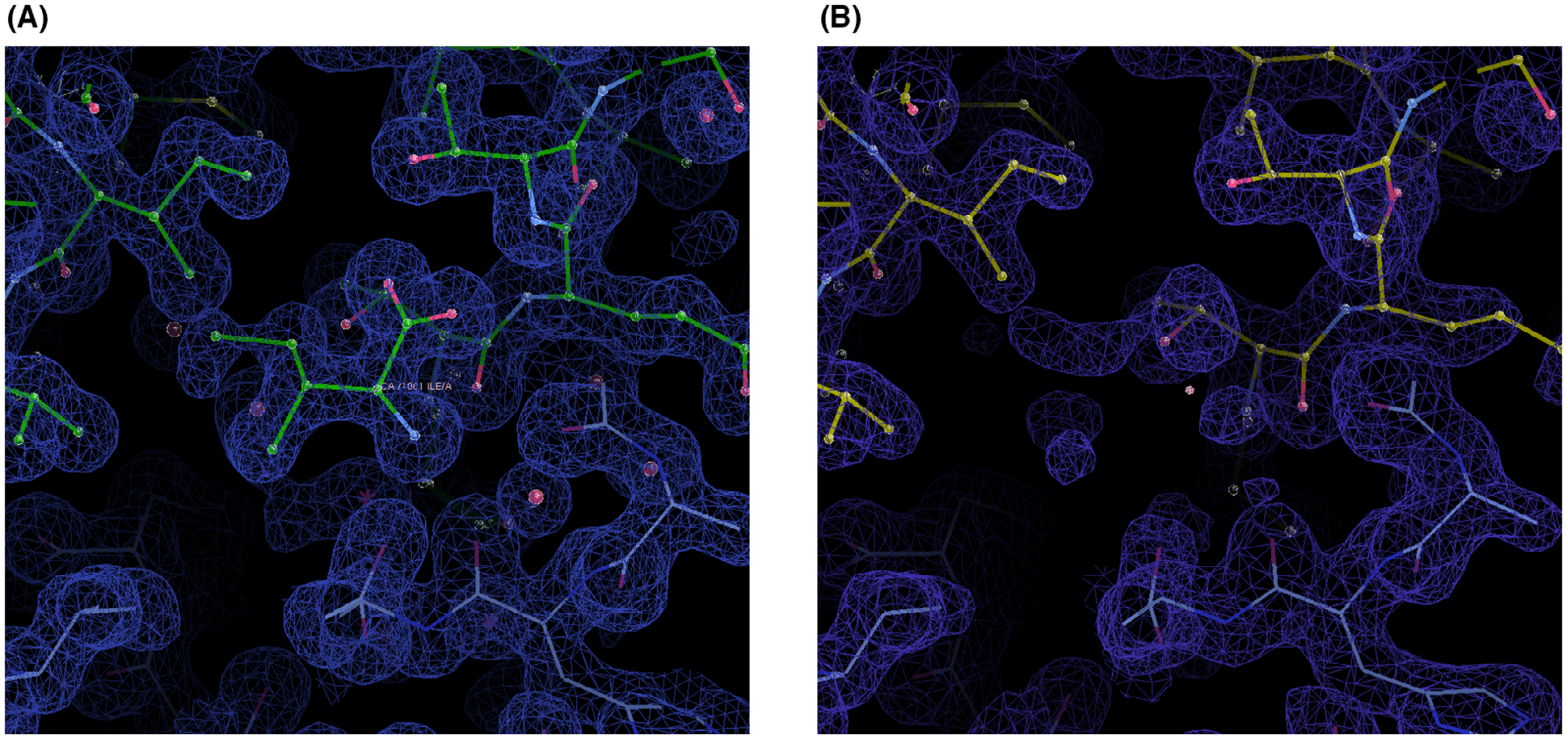
Electron density maps of His‐tagged and thrombin (Tb)‐digested d‐amino acid‐responsive regulatory proteins (DARPs). Electron density maps of the Ile‐binding sites of His‐tagged DARP (A) and Tb‐digested DARP (B). The l‐Ile molecule and residues in subunit A of His‐tagged DARP (A) are shown in green, whereas the corresponding residues in the same subunit of Tb‐digested DARP (B) are shown in yellow. Symmetry‐related residues in both figures are shown in gray. The 2Fo‐Fc electron density maps are shown as blue mesh contoured at 1.0 σ. Tb‐digested DARP was crystallized in a buffer containing 2.0 m ammonium sulfate and 6% (v/v) 2‐propanol as described in the text. An electron density map of the crystal was obtained using the methods described in the Experimental procedures section. Molecular graphics were generated with Coot [26].

## Discussion

### Postulated expression mechanism of BAR and *PH0137* genes

This study found that in *P. horikoshii*, the *PH0138* and *PH0137* genes are simultaneously expressed in the presence of d‐*allo*‐Ile when l‐Ile is absent, but not when other l‐amino acids are limited; l‐Ile represses its expression. Gene expression levels increased significantly during the first hour, then decreased to approximately 50% of the original level. This observation suggests that l‐Ile, which is ultimately synthesized by BAR, mitigates this effect. Therefore, it is necessary to determine the concentrations of l‐Ile and d‐*allo*‐Ile in *P. horikoshii* cells grown under each condition. The *PH0140* gene also forms part of the BAR cluster, but it was not induced in the analysis, suggesting that DARP controls the *PH0137* and *PH0138* genes via mechanisms distinct from those of other FFRPs. Thus, *P. horikoshii* might utilize l‐Ile as a strategic amino acid for adaptation to nutrient fluctuation, in addition to l‐Lys (as described later).

EMSA results showed that DARP specifically bound to the *PH0137* promoter region in the presence of l‐Ile. While direct evidence is lacking, it appears that when l‐Ile is present, DARP binds to the *PH0137* promoter and likely represses the transcription of the *PH0137* and *PH0138* genes. In contrast, when d‐*allo*‐Ile is available, DARP releases from DNA, allowing transcription to proceed.

The PH0137 protein was identified via BLAST (https://blast.ncbi.nlm.nih.gov/Blast.cgi) as a probable member of the YkvI transporter family. Additionally, analysis of the SOSUI program (https://harrier.nagahama‐i‐bio.ac.jp/sosui/) [27] indicated that it is a membrane protein possessing 10 transmembrane regions. These findings suggest that PH0137 may act as an amino acid transporter, facilitating the import of d‐amino acids into the cell. Accordingly, the following scenarios are possible (Fig. 9): (a) When l‐Ile is largely present in the environment (feast mode) and available for the cells, it binds to DARP. The complexed form of the regulatory protein subsequently binds the *PH0137* promoter, repressing *PH0137* and *PH0138* expression. (b) When l‐Ile is depleted, and d‐*allo*‐Ile is present in the environment (famine mode), d‐*allo*‐Ile is taken up by the cell through the PH0137 protein expressed at a basal level. (c) The binding of d‐*allo*‐Ile to DARP causes DARP to be released from the *PH0137* promoter, effectively turning on the d‐amino acid utilization switch via transcription of the *PH0137* and *PH0138*. However, the environmental origin of d‐*allo*‐Ile remains unclear. Further information will become available in the future.

**Fig. 9:**
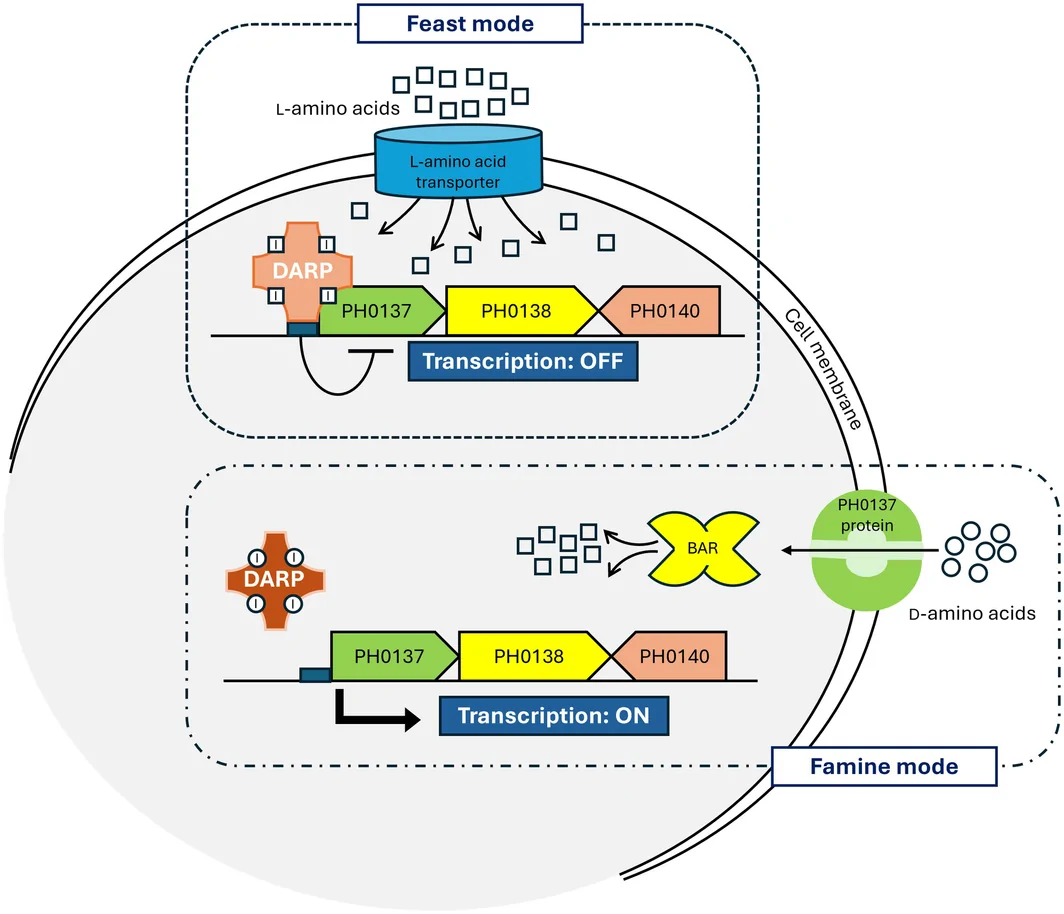
Proposed regulatory mechanism of the broad substrate specificity amino acid racemase (BAR) gene cluster. In feast mode, l‐amino acids (squares) in the medium are incorporated into cells via l‐amino acid transporter. Under these conditions, d‐amino acid‐responsive regulatory protein (DARP) selectively binds to l‐Ile. l‐Ile‐bound DARP binds to *PH0137* promoter, repressing *PH0137* and *PH0138* expression. In the absence of l‐Ile (famine mode), d‐amino acids (circles) in the medium are incorporated into cells via the PH0137 protein (putative d‐amino acid transporter). When d‐*allo*‐Ile binds to DARP, DARP is released from the *PH0137* promoter, effectively turning on the transcription of the *PH0137* and *PH0138* genes. Overexpressed BAR and PH0137 proteins accelerate the incorporation of d‐amino acids and racemization to l‐amino acids.

The BAR cluster containing the DARP was exclusively present in *P. abyssi*, *Pyrococcus* sp. NA2, and *P. horikoshii*. DARP homologs PAB2257 and PNA2_0668 show approximately 90% similarity to *P. horikoshii*'s DARP, suggesting possible responsiveness to d‐amino acids. However, this has not been experimentally validated. Hence, future studies should employ *in vitro* DNA‐binding or reporter‐based transcriptional assays with various d‐amino acids, as well as structural analyses to identify the molecular determinants of ligand specificity.

### Structural insight into coregulator‐binding modes

The structures of *P. horikoshii* FL11 in complex with a DNA duplex (PDB code: 2E1C) and in the DNA‐unbound form (PDB code: 1RI7) have been reported [28, 29]. Comparison of the FL11 dimer in the DNA‐bound and DNA‐unbound forms showed that the distance between the two subunits in the former was larger than that in the latter. This open form may maintain DNA binding in the bent conformation (Fig. 10A). In DNA‐bound structures, Leu24, Arg25, Ala34, Glu35, Ser36, Thr37, His39, and Arg41 make chemical contacts with DNA through hydrogen bonds or hydrophobic interactions [29]. We observed that the distances between the Cα atoms of these residues were reduced in the DNA‐unbound (closed) dimer compared with the DNA‐bound (open) dimer: 0.51–2.23 Å (Fig. 10A). In the DARP structure, Glu35, Ser36, Thr37, and Arg41 in FL11 corresponded to Glu42, Ser43, Thr44, and Arg48, respectively. Although Leu24, Arg25, Ala34, and His39 were replaced with Tyr31, Ser32, Pro41, and Arg46, respectively, the Cα atoms of the corresponding residues occupied similar positions. Among these residues, the interatomic distances between the Cα atoms in the dimer were all reduced in the d‐*allo*‐Ile‐bound structure compared with the l‐Ile‐bound structure (reductions: 0.28–1.71 Å) (Fig. 10B). These observations indicate that the dimeric arrangement of l‐Ile‐bound DARP resembles that of the DNA‐bound form of FL11 (open form), whereas that of d‐*allo*‐Ile‐bound DARP corresponds to the DNA‐unbound form (closed form).

**Fig. 10:**
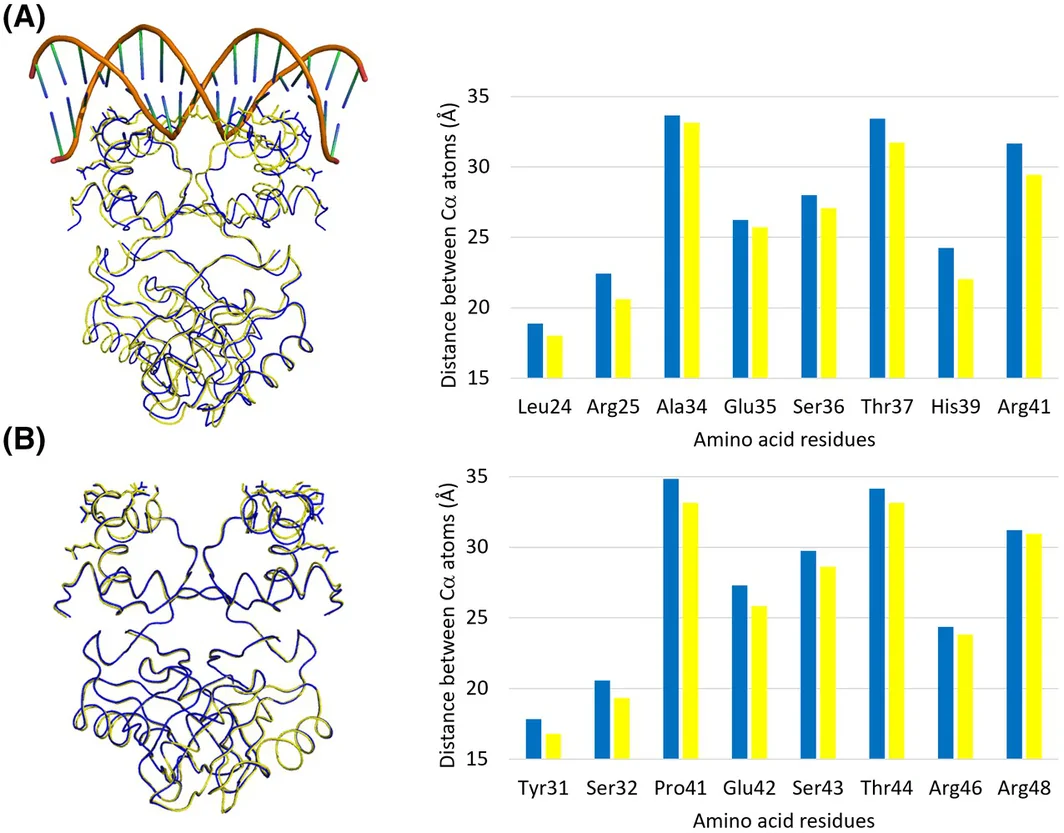
Comparison of FL11 and d‐amino acid‐responsive regulatory protein (DARP) dimer structures. (A) Comparison of the FL11 dimer in the DNA‐bound form (blue) with that of the DNA‐unbound form (yellow) (left). Residues that form chemical contacts with DNA are shown as stick models: the interatomic distances between the Cα atoms of the same residues in the dimer are shown in a bar chart (blue: DNA‐bound form, yellow: DNA‐unbound form) (right). (B) Comparison of the DARP dimer structure of the l‐Ile‐bound form (blue) with that of the d‐*allo*‐Ile‐bound form (yellow) (left). Residues corresponding to the FL11 residues forming chemical contacts with DNA are shown as stick models, and the interatomic distances between the Cα atoms of the same residues in the dimer are shown in a bar chart (blue: l‐Ile‐bound form, yellow: d‐*allo*‐Ile‐bound form) (right). Molecular graphics were generated with PyMOL (http://www.pymol.org/).

Superimposing subunit A of the l‐Ile‐bound octamer onto that of the d‐*allo*‐Ile‐bound octamer revealed that, in the d‐*allo*‐Ile‐bound structure, the DNA‐binding domain of the neighboring subunit (subunit G) was shifted in the opposite direction from subunit A (Fig. 11). Because the same movement was observed in the other subunit of the dimer, the DNA‐binding domain of the d‐*allo*‐Ile‐bound dimer adopted a closed conformation. Interestingly, subtle alterations in the hydrogen bonds around the coregulators caused extensive conformational changes.

**Fig. 11:**
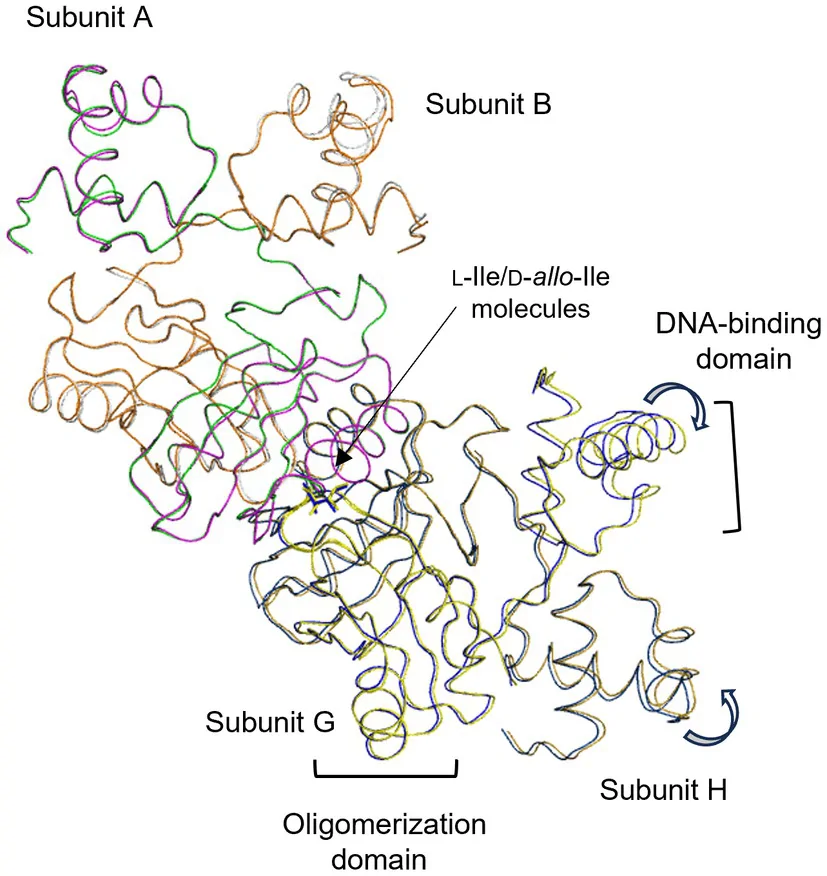
Conformational change in neighboring subunits. Structure of subunit A (green) of the l‐Ile‐bound octamer is superimposed onto that of the d‐*allo*‐Ile‐bound octamer (magenta). The neighboring subunits (subunit G) in the l‐Ile‐bound octamer and the d‐*allo*‐Ile‐bound octamer are shown in blue and yellow, respectively. Subunits B and H in the l‐Ile‐bound octamer are shown in orange and navy, respectively, and the corresponding subunits in the d‐*allo*‐Ile‐bound octamer are shown in gray and gold. The l‐Ile and d‐*allo*‐Ile molecules are shown as stick models in blue and yellow, respectively. Molecular graphics were generated with PyMOL (http://www.pymol.org/).

### Functional difference between DARP and FL11

FFRPs are major transcription factors in archaea and are homologs of the leucine‐responsive regulatory protein (Lrp) in *E. coli* [30, 31, 32]. By sensing rich nutrition through high concentrations of l‐Leu [33], *E. coli* shifts its metabolism from famine to feast mode, thereby regulating 40–70 transcription units [34, 35]. To summarize this global regulation, the term feast/famine regulation was introduced [34], and the Lrp homologs are referred to as “feast/famine regulatory proteins (FFRPs).” Fourteen FFRPs have been reported in *P. horikoshii* to date [21, 32]. They are divided into 11 full‐length types (FL1–FL11), which contain both DNA‐binding and assembly domains, and three demi types (DM1–DM3), which lack the DNA‐binding domain [28]. FL11 comprises 151 amino acids, has a molecular mass of approximately 18 kDa, and primarily regulates various metabolic processes, such as ATP synthesis, transmembrane transport, and DNA synthesis by altering the association states of the dimer and octamer in response to l‐Lys concentrations in the cells [21]. In nutrient‐rich conditions with abundant l‐Lys, FL11 forms an octamer through the association of four dimers [36]. This octamer preferentially binds to the *fl11* promoter and inhibits its transcription. The consequent decrease in FL11 levels releases the repression of roughly 200 transcriptional units, thereby stimulating metabolic activity [29]. When l‐Lys becomes scarce under famine conditions, the octamer dissociates into dimers [36]. This change weakens repression of *fl11* transcription, allowing FL11 dimers to accumulate. Elevated levels of FL11 dimers then repress the transcription of many genes, leading to growth arrest [29].

The DARP is an FFRP designated FL5 [21] and has the highest sequence identity (38%) with FL11 among the FFRP homologs. However, the function of DARP observed in the present study differed from that of FL11. The expression of genes other than *PH0138* and *PH0137* did not change in the presence or absence of d‐*allo*‐Ile (Table 1). This indicated that DARP, unlike FL11, regulates a limited number of genes. Furthermore, FL11 regulates its own expression by binding the *fl11* promoter, whereas DARP binds to the *PH0137* promoter but does not regulate its own expression. Additionally, the association and dissociation of oligomeric structures observed in FL11 do not likely occur in DARP (Fig. 4E) [36].

l‐Phe, l‐Ile, l‐Leu, l‐Val, and l‐Met have been reported as potential ligands for FL5 [21]. Although weaker than that of l‐Ile, the DNA‐binding activity of DARP was also observed in the presence of the l‐isomers of Leu, Met, and Val and was suppressed in the presence of their d‐isomers (Fig. 5C,D). This suggested that *PH0138* and *PH0137* expression could also be induced by culturing *P. horikoshii* with these d‐amino acids. When *P. horikoshii* was grown in the absence of l‐Ile and induced with d‐Val for 1 h, BAR gene expression increased 2.5‐fold (data not shown). In addition, the specific activity of BAR in the crude extracts increased 15‐fold when induced with d‐Val for 8 h (data not shown). Amino acid concentrations used in the cultivation experiments were 0.5–1.3 mm. In contrast, amino acid concentrations are below μm levels, and d/l ratios of Asp, Glu, and Ala are quite low in water samples from deep‐sea hydrothermal vents [37]. Several hyperthermophilic archaea including *P. horikoshii* exhibit no or weak growth due to the lack of hydrophobic and aromatic amino acids in the medium [19], suggesting they require a nutrient‐rich environment for growth. Although investigating the source of d‐amino acids in these environments is important, racemic amino acids are believed to form in deep‐sea hotspots where *P. horikoshii* was found [37]; therefore, these organisms may have developed an on/off switch mechanism for d‐amino acids utilization via DARP. This represents a novel gene expression mechanism in d‐amino acid metabolism regulated by an FFRP homolog.

Cumulatively, the results of this study provide novel insights into the physiological roles of d‐amino acids and their metabolic enzymes in archaeal cells. While this study provides a potential mechanistic explanation for d‐*allo*‐Ile–dependent regulation, its physiological significance is not yet clear and warrants additional investigation under natural environmental conditions.

## Experimental procedures

### Growth of *P. horikoshii*

*P. horikoshii* OT‐3 strain was grown at 90 °C for 18–24 h under anaerobic conditions. Standard medium containing tryptone and yeast extract was used for pre‐pre‐cultivation and defined medium was used for pre‐ and main cultivation, as previously described [19]. Yeast extract (0.1 g/L) was added to the medium without l‐Ile. Cultured cells were collected by centrifugation (7000 × ***g***, 10 min) washed twice with 3% NaCl, and used for subsequent experiments.

### Determination of the BAR activity and protein concentration

*P. horikoshii* cells were suspended in 10 mm HEPES (pH 7.5), and crude extracts were prepared by sonication. BAR activity was spectrophotometrically assayed as previously described using a U‐2900 spectrophotometer (Hitachi, Tokyo, Japan) [19]. In this assay, l‐Met was routinely used as the substrate. Protein concentration was determined using a Protein Assay (Bio‐Rad) with bovine serum albumin as the standard.

### RNA‐seq analysis

*P. horikoshii* was cultivated in defined medium containing 20 types of l‐amino acids (24 h) and in a substitution medium in which l‐Ile was replaced with d‐*allo*‐Ile (24 h) (d‐*allo*‐Ile substitution medium). Cells collected from 400 mL of culture were used for total RNA extraction with the RNeasy Mini kit (Qiagen, Tokyo, Japan). Subsequently, ribosomal RNA in 1 μg of total RNA was removed using the NEBNext rRNA Depletion Kit (New England Biolabs, Tokyo, Japan). cDNA was synthesized with a TruSeq Stranded mRNA Library Prep Kit (Illumina, Tokyo, Japan) and analyzed using a NextSeq 500 (Illumina). RNA‐seq reads were trimmed with the CLC Genomics Workbench 11 (Qiagen). Gene expression levels were calculated as TPM.

### RT‐qPCR

For RT‐qPCR, after extracting total RNA, cDNA was synthesized using ReverTra Ace qPCR RT Master Mix (Toyobo, Tokyo, Japan). The reaction mixture (10 μL) containing 5 × RT Master Mix and 1 μg of total RNA that had been preincubated at 60 °C for 5 min was prepared, and reverse transcription and enzyme denaturation were performed as follows: 37 °C for 15 min, 50 °C for 5 min, and 98 °C for 5 min using a thermal cycler (Bio‐Rad).

qPCR analysis was performed with KOD SYBR qPCR Mix (Toyobo). The reaction mixture containing 2 × qPCR Master Mix, 0.2 μm each primer set (Table 3), and cDNA in a total volume of 20 μL was prepared, and the reaction was performed as follows: one cycle of 98 °C for 2 min and 40 cycles of 98 °C for 10 s, 60 °C for 10 s, and 68 °C for 30 s. Specific amplification of the target was confirmed by melting curve analysis. 23S rRNA was used as the reference gene for normalization of gene expression. Primer sequences were designed using Primer‐BLAST (https://www.ncbi.nlm.nih.gov/tools/primer‐blast/) to amplify the 100–150 bp of fragment. Three different cDNA concentrations were used to confirm PCR efficiency.

**Table 3:** List of primers used in this study.

Name	Sequence	Purpose
PH0140‐NdeI	ACATATGATAACCGTGCCACACTCAAG	Expression
PH0140‐BamHI	CGGATCCCTAAAACACTCTAGCATATC	Expression
PH0137(−200 ~ +15)‐Fw	TAATCAGCTCAATCATTATAACACTTTGC	EMSA
PH0137(−200 ~ +15)‐Rv	ATGCCCATTACTCATCTTCCTACCTC	EMSA
PH0138(−200 ~ +15)‐Fw	CCACAATCATAATAATCCTTGCAATAGC	EMSA
PH0138(−200 ~ +15)‐Rv	ATCCCACTTGGTCATGGAAACCACC	EMSA
PH0134(−200 ~ +15)‐Fw	AGACGTCTCTAAACTCTATCATCAG	EMSA
PH0134(−200 ~ +15)‐Rv	AACTTTCACTTGCATATTCCTTCCC	EMSA
PH0140(−200 ~ +15)‐Fw	CCTTAATTATGCTTATACTAATTCCTGG	EMSA
PH0140(−200 ~ +15)‐Rv	TGGCACGGTTATCACCTGTATATCTAG	EMSA
PHrRNA02‐qPCR‐Fw	GGAAAGGAAAGTGGCCAAGC	qPCR
PHrRNA02‐qPCR‐Rv	GATCTCGCTCACCTCACCAC	qPCR
PH0137‐qPCR‐Fw	CTCGGCCCTATTCGTTGGAG	qPCR
PH0137‐qPCR‐Rv	CGGGACTGGAGCATTCACAA	qPCR
PH0138‐qPCR‐Fw	ACGCTGGGGTTGTTATACCG	qPCR
PH0138‐qPCR‐Rv	CTCCCCATTCCCGTCTGAAC	qPCR
PH0140‐qPCR‐Fw	CCGTGCCACACTCAAGAAAAG	qPCR
PH0140‐qPCR‐Rv	ATTAACCCTGGCCCTTACCG	qPCR

### Expression and purification of DARP and digestion with thrombin

DARP was produced in recombinant *E. coli*. The *PH0140* gene was PCR‐amplified from *P. horikoshii* genomic DNA using *PH0140*‐NdeI and *PH0140*‐BamHI primers (Table 3) and cloned into the pET11a and pET15b vectors. *E. coli* BL21 (DE3) CodonPlus RIPL cells harboring pET15b/*PH0140* were cultivated in Luria‐Bertani medium containing 50 μg/mL ampicillin, and gene expression was induced for 3 h with 0.5 mm isopropyl‐β‐d‐thiogalactopyranoside. After centrifugation at 5000 × ***g*** for 10 min, the cells were suspended in 10 mm Tris/HCl (pH 8.0), treated with lysozyme, and lysed by sonication. After centrifugation at 15000 × ***g*** for 10 min, the crude extract was heated at 90 °C for 30 min and centrifuged again to remove precipitates. The supernatant was purified using Ni^2+^‐chelating Sepharose with stepwise imidazole elution. The purified preparation was desalted with 10 mm Tris/HCl (pH 8.0), concentrated by ultrafiltration using Amicon Ultra (MWCO 10000) (Millipore, Tokyo, Japan), and used for crystallization. About 10 mg of purified protein was obtained from 1 L of culture.

### EMSA

For EMSA, 1 mg of the purified protein was digested with 1 U thrombin in 1 mL reaction buffer (20 mm Tris/HCl [pH 8.0], 150 mm NaCl, and 2.5 mm CaCl_2_) at 20 °C for 16 h. Digestion was confirmed by SDS/PAGE, followed by desalting and concentration using 10 mm Tris/HCl (pH 8.0).

DNA probes were prepared by PCR using the primer sets listed in Table 3. Each primer set was designed to amplify a 200‐bp region upstream and a 15‐bp region downstream of the start sites of *PH0134, PH0137*, *PH0138*, and *PH0140*. The amplified DNA probes were purified with the Nucleospin Gel and PCR Clean‐up Kit (Macherey‐Nagel). For the assay, 25, 50, or 100 nm DARP (as octamer) was incubated with 10 nm DNA probe at 70 °C for 15 min in binding buffer (10 mm Tris/HCl [pH 7.0], 1 mm DTT, and 400 mm KCl) in the presence or absence of d‐ or l‐amino acids. After incubation, the samples were loaded onto a 5% polyacrylamide gel, and electrophoresis was performed using 0.5 × TBE buffer at 100 V for 60 min. After electrophoresis, gels were stained with ethidium bromide. Band shifts were detected using a UV illuminator.

### Crystallization and data collection

His‐tagged DARP was used for crystallization analyses. Crystals of l‐Ile‐bound DARP were obtained using the sitting‐drop vapor diffusion method, in which a 1.5 μL drop of 10.4 mg/mL protein solution was mixed with an equal volume of mother liquor composed of 0.1 m sodium cacodylate‐HCl buffer (pH 6.5) and 1.26 m ammonium sulfate. For the crystallization of d‐*allo*‐Ile‐bound DARP, a protein solution containing 5 mm d‐*allo*‐Ile was incubated at 80 °C for 20 min. The crystals were grown in sitting drops comprising 1.5 μL heat‐treated protein solution (10.9 mg/mL) and 1 μL mother liquor containing 2.0 m ammonium sulfate and 6% (v/v) 2‐propanol. In both cases, the sitting drops were equilibrated against 0.1 mL of mother liquor, and the crystals were grown for 1 week at 20 °C.

Data were collected using a Dectris Pilatus3 S2M detector system on the AR‐NW12A beamline for l‐Ile‐bound DARP and a Dectris Pilatus 2 M‐F detector system on the AR‐NE3A beamline for d‐*allo*‐Ile‐bound DARP at the Photon Factory, Tsukuba, Japan. Measurements were performed on crystals cooled to 100 K in a stream of liquid nitrogen gas. Cryoprotection was performed with mother liquor containing 30% (v/v) glycerol for l‐Ile‐bound DARP and 30% (v/v) ethylene glycol for d‐*allo*‐Ile‐bound DARP. Data were processed using HKL2000 [38] for l‐Ile‐bound DARP and with XDS [39] for d‐*allo*‐Ile‐bound DARP.

### Phasing, refinement and structure analysis

The structure of the l‐Ile‐bound DARP was solved at a resolution of 1.39 Å by molecular replacement using MOLREP [40] in the CCP4 program suite [41]. The monomer structure predicted with AlphaFold2 [42] was used as the search model. Data in the resolution range of 50–3 Å were used for molecular replacement. The model was built with Coot [26]. Maximum‐likelihood refinement at 1.39 Å resolution was performed with REFMAC5 [43]. After several inspection cycles of the 2Fo‐Fc and Fo‐Fc density maps, the model was rebuilt. The structure of d‐*allo*‐Ile‐bound DARP was solved at a resolution of 2.20 Å by molecular replacement with MOLREP [40]. The structure of l‐Ile‐bound DARP was used as the search model. Data in the resolution range of 50–3 Å were used for molecular replacement. The model was then rebuilt using Coot [26], and refinement at maximum resolution was performed with REFMAC5 [43], as described for l‐Ile‐bound DARP. In all cases, water molecules were incorporated with Coot [26] and model geometry was analyzed with MolProbity [44]. Data collection and refinement statistics are presented in Table 2.

The crystal structure of the *P. horikoshii* PH0140 protein (DARP) bound to l‐Ile has been deposited in the PDB (PDB code: 7FBY, released July 20, 2022); however, the detailed discussion based on this structure has not been reported to date.

## Author contributions

RK, HS, and TO designed the research and provided scientific guidance; TK performed the cultivation of *P. horikoshii*, enzyme activity assays, and gene expression analysis; TK and TU performed EMSA analysis and crystal preparation; HS and JH collected X‐ray diffraction data and analyzed the structural data; TM provided technical advice for EMSA and RNA‐seq analyses. RK wrote the paper; and RK, HS, TM, and TO revised the paper.

## Conflict of interest

The authors declare no conflict of interest.

## Data Availability

The atomic coordinates and structure factors (PDB: 9W4W for l‐Ile‐bound DARP and 9W4V for d‐*allo*‐Ile‐bound DARP) were deposited in the Protein Data Bank (http://www.rcsb.org/). RNA‐seq data were deposited in DDBJ (https://www.ddbj.nig.ac.jp/) under accession number PRJDB35890. Other data generated and/or analyzed during this study are available from the corresponding authors upon reasonable request.
